# Hypoxia activates IKK–NF-κB and the immune response in *Drosophila melanogaster*

**DOI:** 10.1042/BSR20140095

**Published:** 2014-07-29

**Authors:** Daniel Bandarra, John Biddlestone, Sharon Mudie, H. Arno Muller, Sonia Rocha

**Affiliations:** *Centre for Gene Regulation and Expression, College of Life Sciences, University of Dundee, Dundee DD1 5EH, U.K.; †Division of Cell and Developmental Biology, College of Life Sciences, University of Dundee, Dundee DD1 5EH, U.K.

**Keywords:** Cyld, *Drosophila*, IKK, NF-κB, hypoxia, immune response, FIH, factor inhibiting HIF (hypoxia-inducible factor), IAP, inhibitor of apoptosis, IKK, IκB (inhibitor of nuclear factor κB) kinase, IMD, immuno deficient, NF-κB, nuclear factor κB, PHD, prolyhydroxylase, QPCR, quantitative PCR, qRT–PCR, quantitative real-time PCR, siRNA, small interfering RNA, TAK, TGF (transforming growth factor)-β-activated kinase, TNF, tumour necrosis factor, TRAF, TNF receptor-associated factor, VHL, von Hippel Lindau

## Abstract

Hypoxia, or low oxygen availability, is an important physiological and pathological stimulus for multicellular organisms. Molecularly, hypoxia activates a transcriptional programme directed at restoration of oxygen homoeostasis and cellular survival. In mammalian cells, hypoxia not only activates the HIF (hypoxia-inducible factor) family, but also additional transcription factors such as NF-κB (nuclear factor κB). Here we show that hypoxia activates the IKK–NF-κB [IκB (inhibitor of nuclear factor κB)–NF-κB] pathway and the immune response in *Drosophila melanogaster*. We show that NF-κB activation is required for organism survival in hypoxia. Finally, we identify a role for the tumour suppressor Cyld, as a negative regulator of NF-κB in response to hypoxia in *Drosophila*. The results indicate that hypoxia activation of the IKK–NF-κB pathway and the immune response is an important and evolutionary conserved response.

## Abstract 2

Here we demonstrate that low oxygen activates the transcription factor, NF-κB and the immune response in *Drosophila*. We show that deregulation of the NF-κB response following low oxygen, results in decreased survival of these animals.

## INTRODUCTION

Hypoxia is an important stimulus involved in both physiological and pathological processes [1]. Appropriate response to hypoxia, is required for correct embryo development in mammalian systems (reviewed in [2]), but not for other multicellular organisms such as *Caenorhabditis elegans* [3] and *Drosophila* [4]. However, all of these organisms require the component of the hypoxia response to survive under low oxygen conditions [2–4]. In pathological conditions, such as cancer, hypoxia contributes to the pathology as well as treatment resistance of the disease [5].

At the molecular level, cells respond to hypoxia via the action of a family of transcription factors known as HIFs (hypoxia-inducible factors). HIFs are heterodimers composed of an oxygen-controlled subunit HIF-α, and an oxygen-insensitive partner called HIF-1β [also known as ARNT (aryl hydrocarbon receptor nuclear translocator)] [6]. In mammalians, three isoforms for HIF-α exist, as well as several splice variants for HIF-1β [6]. In organisms, such as the fruit fly, *D. melanogaster*, only one HIF-α exists, called Sima, whereas its binding partner Tango is the *Drosophila* homologue of HIF-1β [4,7].

Oxygen regulation of this transcription factor family is conferred by a family of dioxygenases, called PHDs (prolyl hydroxylases) and FIH (factor-inhibiting HIF) [8]. PHD-mediated hydroxylation of HIF-α subunits creates a high affinity-binding site for the tumour suppressor VHL (von Hippel Lindau) protein, which is part of an ubiquitin E3–ligase complex. VHL-dependent ubiquitination of HIF-α results in rapid proteasomal degradation of HIF-α subunits. FIH-dependent hydroxylation, on the other hand, prevents binding of co-activators to the HIF complex, and hence reduces HIF transcriptional activity [9]. The mechanism of oxygen-dependent degradation of HIF-α is also conserved in *Drosophila*, albeit more simplified, with only one PHD being present (Fatiga) and no FIH homologue being thus far identified [10].

Apart from activation of the HIF system, hypoxia also activates a variety of other transcription factors in mammalian cells and organisms. One important transcription factor activated by hypoxia are members of the NF-κB (nuclear factor κB) family. NF-κB is well known for its role in mediating innate immunity and controlling a variety of survival pathways in the cell [11]. In mammalian systems, NF-κB is composed of five genes, encoding RelA/p65, RelB, cRel, p100/p52 and p105/p50. NF-κB targets are extensive and include genes from many different pathways such as proliferation, apoptosis, inflammation, migration and cell cycle [12].

Given NF-κB's importance, its activation is under precise control and it is very intricate, with numerous feedback mechanisms in place. The best understood activation pathway for NF-κB is following the exposure to the pro-inflammatory cytokine TNF-α (tumour necrosis factor-α). Upon ligand binding to the receptor, adaptor molecules, kinases and ubiquitin ligases are recruited leading to the activation of the TAK [TGF (transforming growth factor)-β-activated kinase]–TAB (TAK-associated binding protein) complex [13]. TAK is essential for the activation of the IKK [IκB (inhibitor of nuclear factor κB) kinase) complex, which in mammalian cells is composed by IKKα, IKKβ and IKKγ [14]. IKK activation leads to phosphorylation of the IκB (inhibitor of κB) proteins, which are instrumental in sequestering NF-κB dimers in the cytoplasm. Upon phosphorylation, IκB proteins are ubiquitinated and degraded by the proteasome, thus releasing NF-κB to translocate to the nucleus, where it binds κB-sites in the promoters and enhancers of its target genes [15].

Many negative feedback points exist in the NF-κB pathway [16]. These are designed to optimal control of the activity of this transcription factor. Interestingly, many of these negative regulators are mutated in human disease [12]. These include IκBs, A20 and Cyld two deubiquitinating enzymes [17]. Cyld is a known tumour suppressor [18] and it is thought to act at the level of adaptor molecules such as TRAFs (TNF receptor-associated factor) and the IKK complex itself, namely on IKKγ ubiquitination [18].

NF-κB's function in *Drosophila* has been extensively studied and it has been shown to be essential for innate immune responses in this organism. In *Drosophila*, NF-κB is composed of three members: Dorsal, Dif and Relish. The activation pathway has also been well characterized and it is well conserved in these animals. Homologues for IKK (Ird5 and Kenny) and TAK exist, and these have been shown to be required for activation of NF-κB/Relish following infection [19]. In addition, negative regulators such as IκB (cactus) and Cyld also are present in this organism [19].

Activation of the NF-κB pathway in *Drosophila* can occur via the Toll or the IMD (immuno deficient) pathways depending, for the most part, on the type of pathogen [19]. While the activation of the Toll pathway leads to cactus degradation and release of Dorsal and Dif, activation of the IMD pathway, requires dTAK1, dIKKβ (Ird5) and culminates in the processing and nuclear accumulation of active Relish [19]. Dorsal, Dif and Relish induce the expression of a variety of anti-immune peptides and proteins that help defend the organism against infection [19].

In this study, we investigated whether hypoxia activates IKK–NF-κB in *Drosophila* as it occurs in mammalian cells. We show that hypoxia activates NF-κB in an IKK-dependent manner, and that hypoxia-triggered NF-κB-dependent gene expression is induced both in larvae and adult flies. We find that Cyld acts as a negative regulator of this pathway in response to hypoxia. Finally, we show that precise control of the NF-κB response in flies is required for survival in hypoxia in this model organism.

## EXPERIMENTAL

### Cells

Human cervix carcinoma HeLa, and human osteosarcoma U2OS cell lines were obtained from the A.T.C.C. All cells were maintained at 5% (v/v) CO_2_ in DMEM (Dulbecco's modified Eagle's medium) (Lonza) supplemented with 10% (v/v) FBS (fetal bovine serum) (Invitrogen), 1% penicillin–streptomycin (Lonza) and 1% L-glutamine (Lonza).

### Treatments

Hypoxia treatments were performed in an InVIVO 300 hypoxia workstation (Ruskin). All extracts were performed inside the workstation to avoid re-oxygenation.

### Antibodies

Antibodies against HIF-1α were obtained from BD Biosciences; p-RelA (536 and 468), A20, IKKβ and β-actin were from Cell Signalling; RelA from Santa Cruz Biotechnology; IAP1 (inhibitor of apoptosis 1) from R&D systems. ECL (enhanced chemiluminescence, Pierce) was used for detection.

### *Drosophila* strains, protein and mRNA extracts preparation

Fly culture and husbandry were performed according to standard protocols. Homozygous flies carrying *Ird5^1^* mutation [20] were used as an IKK loss-of-function model, and *CYLD^f00814^* [21] loss-of-function model. *CYLD* loss-of-function flies were generated by Exelixis and made available to the fly community via the Bloomington Stock Centre. *sima^07607^* [22] flies were used as an HIF-1α loss-of-function model. *White^1118^* flies were used as control. Third-instar larvae or adults were exposed to 5 or 3% O_2_ prior to RNA extraction. mRNA extracts we prepared from adult heads or whole-larvae body using TRIzol (Invitrogen). Gene expression levels of several transcripts were measured by qRT–PCR (quantitative real-time PCR). The primers used for qRT–PCR are shown in Table 1.

**Table 1:** Primers were used for qRT–PCR

Primer name	Forward sequence (5′→3′)	Reverse sequence (5′→3′)
Dractin	GCGTTTTGTACAATTCGTCAGCAACC	GCACGCGAAACTGCAGCCAA
Sima	AGCCCAATCTGCCGCCAACC	TGGAGGCCAGGTGGTGGGAC
dLdh	CAGTTCGCAACGAACGCGCA	CAGCTCGCCCTGCAGCTTGT
Diptericin	ACCGCAGTACCCACTCAATC	ACTTTCCAGCTCGGTTCTGA
Drosocin	CCATCGTTTTCCTGCTGCTTGC	GTCAGGTGATCCTCGATGGCCA
Drosomycin	GTTCGCCCTCTTCGCTGTCCTGA	CCTCCTCCTTGCACACACGACG
Relish	GACCCGAAAGCTCGGCGCAAA	TCGCTCACGAGTTGCGAGCAA
Dorsal	TGTTCAAATCGCGGGCGTCGA	TCGGACACCTTCGAGCTCCAGAA
Dif	CGGACGTGAAGCGCCGACTTG	CAGCCGCCTGTTTAGAGCGG
dCYLD	TGCCTTCCAACTCTCGTCTT	AAGGACGCTGATGAAGGAGA
Attacin A	AGGTTCCTTAACCTCCAATC	CATGACCAGCATTGTTGTAG
Attacin B	GGGTAATATTTAACCGAAGT	GTGCTAATCTCTGGTCATC

### Mammalian mRNA extract preparation and QPCR (quantitative PCR) analysis

Total RNA from mammalian cells was extracted using peqGOLD total RNA kit (Peqlab), according to the manufacturer's directions. RNA was converted to cDNA using Quantitect Reverse Transcription Kit (Qiagen). For QPCR, Brilliant II SYBR green kit (Stratagene/Agilent), including specific MX3005P 96 well semi-skirted plates, were used to analyse samples on the Mx3005P QPCR platform (Stratagene/Agilent). Actin was used as a normalizing gene in all experiments. The Primers used for RT–QPCR are shown in Table 2.

**Table 2:** Primers were used for QPCR

Primer name	Forward sequence (5′→3′)	Reverse sequence (5′→3′)
Actin	CTGGGAGTGGGTGGAGGC	TCAACTGGTCTCAAGTCAGTG
XIAP (X-linked inhibitor of apoptosis)	CTGTTAAAAGTCATCTTCTCTTGAAA	GACCCTCCCCTTGGACC
A20	ACAGCTTTCCGCATATTGCT	TTGACCAGGACTTGGGACTT
IAP1	AACTCTTGGCCTTTCATTCG	TGTTGTGATGGTGGCTTGAG

### siRNA (small interfering RNA), whole-cell protein lysates and Western blotting

siRNA transfections, whole-cell protein lysates and Western blotting were performed essentially as described previously [23].

## RESULTS

### IKK and NF-κB are activated following hypoxia in cancer cells

**Figure 1:**
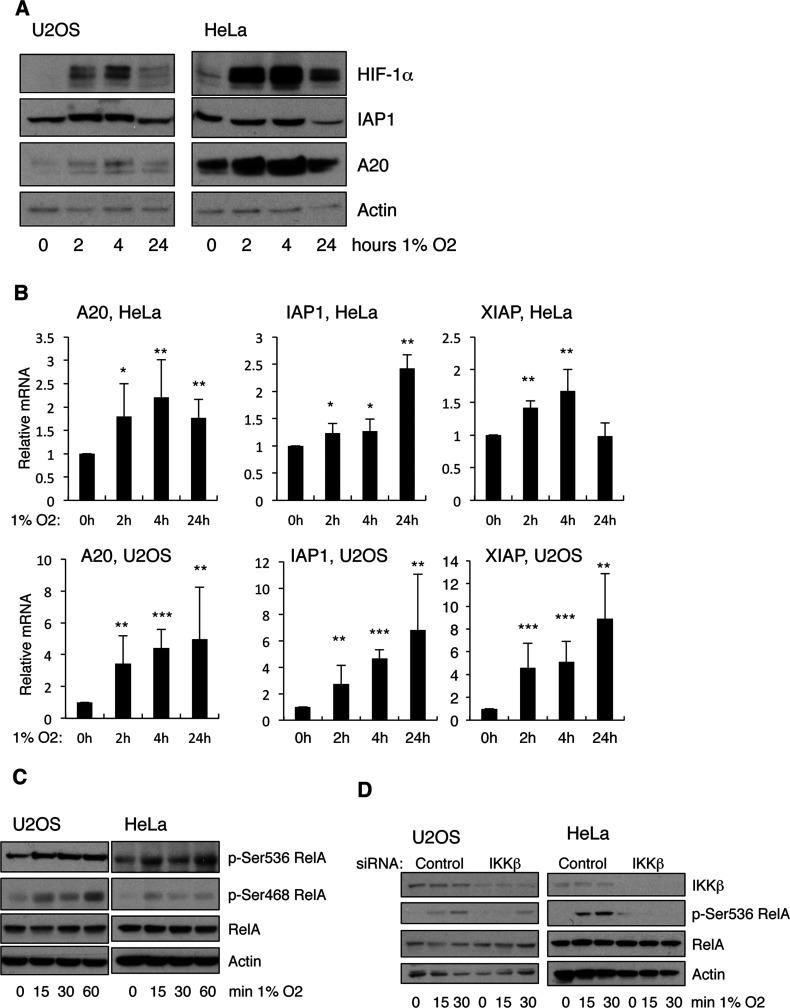
Hypoxia activates IKK-NF-κB in mammalian cancer cells (**A**) U2OS and HeLa cells were exposed to 1% O_2_ for the indicated periods of time prior to lysis. Whole cell lysates were analysed by Western blot using the depicted antibodies. (**B**) U2OS and HeLa cells were treated as in (**A**) but total RNA was extracted. Levels of mRNA for the depicted genes were analysed by QPCR using actin as a normalizing gene. Graph depicts mean and S.D. of a minimum of three independent experiments. Student's *t* test was performed and significance determined as follows:*P≤0.05, **P≤0.01, ***P≤0.001. (**C**). U2OS and HeLa cells were exposed to 1% O_2_ for the indicated periods of time prior to lysis. Whole-cell lysates were analysed by Western blot using the depicted antibodies. (**D**). U2OS and HeLa cells were transfected with control or IKKβ siRNA oligonucleotides prior to exposure to 1% O_2_ for the indicated periods of time. Whole-cell lysates were obtained 48 h post-transfection, and analysed by Western blot using the depicted antibodies.

We have previously shown that hypoxia can lead to activation of the NF-κB pathway, and modulation of NF-κB transcriptional activity in mammalian cells [24,25]. In our studies, we had identified IL-8 (interleukin-8), NF-κB2, and IAP2, as three genes that were induced in hypoxia in a NF-κB-dependent manner [24]. To further understand the significance of NF-κB regulation in hypoxia, we analysed additional NF-κB targets in response to hypoxia. When U2OS and HeLa cells were exposed to 1% O_2_ we could detect an increase in the levels of A20 and IAP1 proteins following 2 and 4 h exposure to 1% O_2_ (Figure 1A). However, this increase was no longer evident following 24 h of hypoxia. The increase in these genes was also evident at the transcriptional level, as QPCR analysis demonstrated an increase in the mRNA of these genes following exposure to hypoxia (Figure 1B).

One mechanism by which NF-κB can be modulated is via post-translational modifications, most prevalently phosphorylation [12]. RelA can be phosphorylated by IKK at several sites, including Ser^536^ and Ser^468^ [15]. Since we had previously shown that hypoxia activates IKK, we analysed if RelA was phosphorylated at these sites in hypoxia using phospho-specific antibodies (Figure 1C). Exposure of U2OS and HeLa cells to short times of hypoxia lead to increased phosphorylation of both Ser^536^ and Ser^468^ residues in RelA, without any changes to the total levels of the protein. Importantly, phosphorylation of Ser^536^ of RelA following hypoxia was significantly reduced in the absence of IKKβ (Figure 1D). These results further validate the activation of IKK under hypoxia.

### Hypoxia activates NF-κB in *Drosophila*

**Figure 2:**
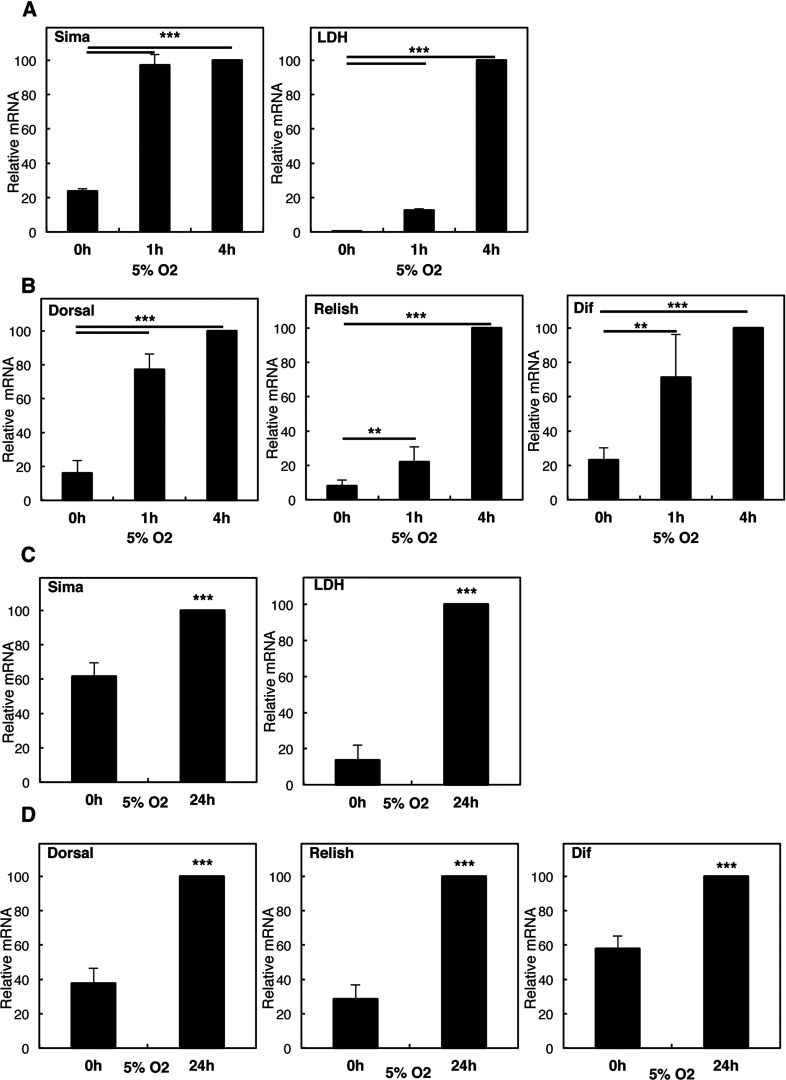
Hypoxia induces increases in NF-κB subunit levels in larvae and adult *Drosophila* (**A**) Third-instar larvae were exposed to 5% O_2_ for the indicated times prior to total RNA extraction. Levels of *Sima* and its target *Ldh* were analysed by QPCR using actin as a normalizing gene. Graph depicts mean and S.D. of a minimum of three independent experiments. Student's *t* test was performed and significance determined as follows:*P≤0.05, **P≤0.01, ***P≤0.001. (**B**) As in (A), but levels of NF-κB subunits Dorsal, Relish and Dif were analysed by QPCR, using actin as a normalizing gene. Graph depicts mean and S.D. of a minimum of three independent experiments. Student's *t* test was performed and significance determined as follows:*P≤0.05, **P≤0.01, ***P≤0.001. (**C**) Adult animals were exposed to 5% O_2_ for 24 h prior to total RNA extraction. Levels of *Sima* and its target *Ldh* were analysed as in (A). Graph depicts mean and S.D. of a minimum of three independent experiments. Student's *t* test was performed and significance determined as follows:*P≤0.05, **P≤0.01, ***P≤0.001. (**D**). Adult animals were treated as in (**C**) and analysed as in (**B**). Graph depicts mean and S.D. of a minimum of three independent experiments. Student's *t* test was performed and significance determined as follows:*P≤0.05, **P≤0.01, ***P≤0.001.

Given our results in the tissue culture cell model, we wanted to validate our results in the context of a whole organism. To this end, we analysed *Drosophila melanogaster*, as a model organism where NF-κB activation and function has been well studied [19]. In larvae, hypoxia induced a time-dependent activation of *Sima* (HIF-1α homologue) and its target *Ldh* (Figure 2A). Interestingly, hypoxia also led to the induction of the NF-κB subunits *dorsal*, *relish* and *dif* (Figure 2B). To rule out the development-specific responses, we also analysed adult animals. Following hypoxia exposure, *sima* was induced at the mRNA levels as previously seen in larvae, and, importantly, hypoxia induced the expression of Sima target gene *Ldh* in adult animals (Figure 2C). As observed in the larvae stage, exposure of *Drosophila* adults to hypoxia also resulted in the induction of mRNA expression of the NF-κB subunits *dorsal*, *relish* and *dif* (Figure 2D). Taken together these results demonstrate that hypoxia induced mRNA expression of NF-κB independent of the developmental stage.

**Figure 3:**
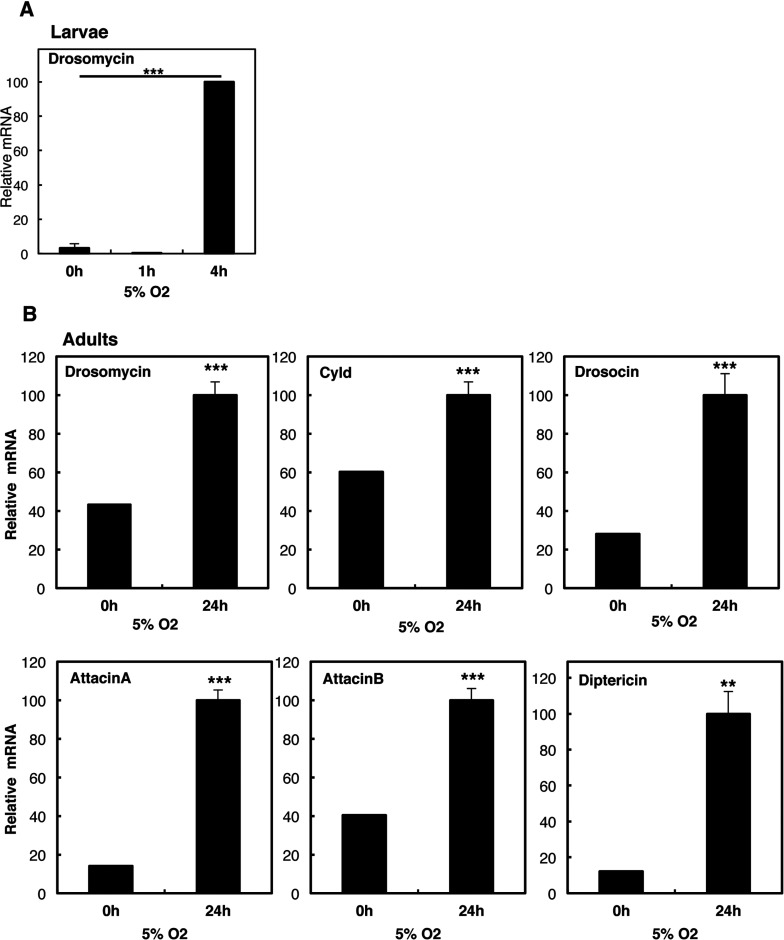
Hypoxia activates the immune response in larvae and adult *Drosophila* (**A**) Third-instar larvae were exposed to 5% O_2_ for the indicated times prior to total RNA extraction. Levels of Drosomycin were analysed by QPCR using actin as a normalizing gene. Graph depicts mean and S.D. of a minimum of three independent experiments. Student's *t* test was performed and significance determined as follows:*P≤0.05, **P≤0.01, ***P≤0.001. (**B**) Adult animals were exposed to 5% O_2_ for 24 h prior to total RNA extraction. Levels of the indicated immune response genes were analysed by QPCR using actin as a normalizing gene. Graph depicts mean and S.E.M. of a minimum of three independent experiments. Student's *t* test was performed and significance determined as follows:*P≤0.05, **P≤0.01, ***P≤0.001.

To determine if elevated NF-κB subunits could result in the induction of NF-κB target genes, we next analysed a panel of known NF-κB-dependent genes in *Drosophila*. While we could observe an increase in the NF-κB target gene *drosomycin* in larvae exposed to hypoxia (Figure 3A), we conducted most of our analysis in adult flies, in order to rule out any developmental effects. Hypoxia treatment of adult animals resulted in the induction of all NF-κB-dependent targets analysed, including *drosomycin*, *drosocin*, *attacin A*, *diptericin* and *cyld* (Figure 3B). These results demonstrate that hypoxia activates NF-κB and the immune peptide response in *Drosophila*.

### Hypoxia activated NF-κB in *Drosophila* is Ird5/IKK dependent

**Figure 4:**
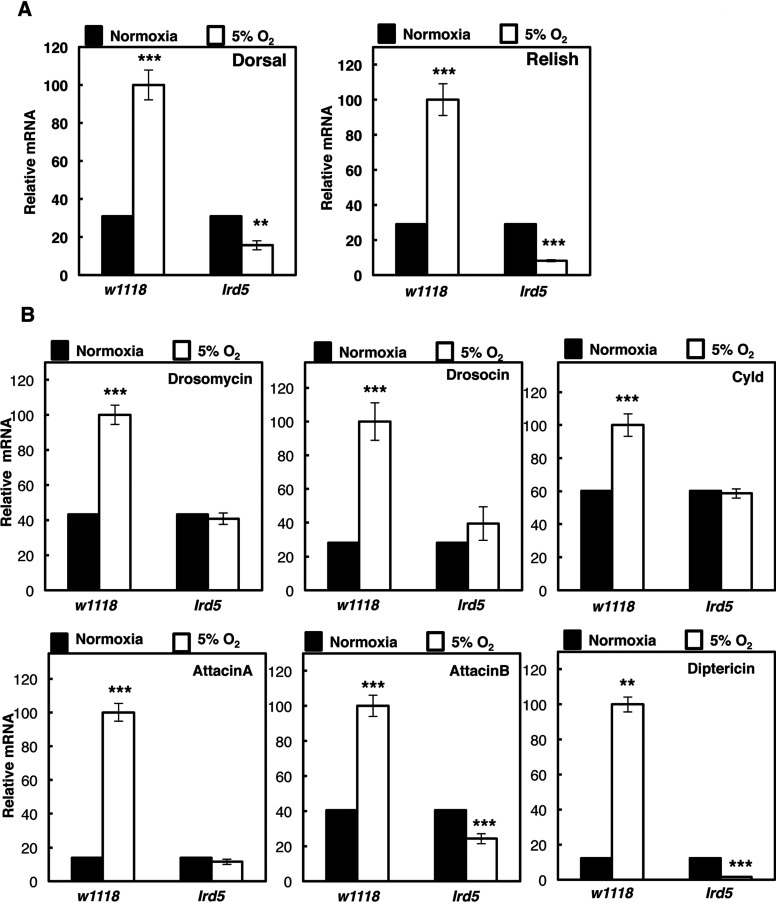
Hypoxia activated NF-κB and immune response in *Drosophila* is IKK (Ird5) dependent (**A**) Wild-type (*w1118*) and IKK-deficient adult animals (*Idr5*) were exposed to 24 h 5% O_2_ prior to total RNA extraction. Levels of Dorsal and Relish mRNA were analysed by QPCR using actin as a normalizing gene. Graph depicts mean and S.E.M. of a minimum of three independent experiments. Student's *t* test was performed and significance determined as follows:*P≤0.05, **P≤0.01, ***P≤0.001. (**B**). Wild-type (*w1118*) and IKK-deficient adult animals (*Idr5*) were exposed to 24 h 5% O_2_ prior to total RNA extraction. Levels of the indicated immune response genes were analysed as in (**A**). Graph depicts mean and S.E.M. of a minimum of three independent experiments. Student's *t* test was performed and significance determined as follows:*P≤0.05, **P≤0.01, ***P≤0.001.

Our mammalian studies had demonstrated that hypoxia activates NF-κB in an IKK-dependent manner [24,25]. To determine whether this is also conserved in the *Drosophila* model, we repeated our analysis in the presence or absence of Ird5 function, the IKK homologue in the fly [19,26]. In the absence of Ird5 function, hypoxia exposure did not result in increased mRNA of both *dorsal* and *relish* (Figure 4A). Importantly, the levels of NF-κB-dependent targets were no longer induced by hypoxia, in the absence of a functional Ird5 (Figure 4B). These results indicate that in *Drosophila*, such as in mammalian systems, hypoxia activates NF-κB in an IKK-dependent manner.

### Hypoxia induced NF-κB gene expression in *Drosophila* is controlled by Cyld

**Figure 5:**
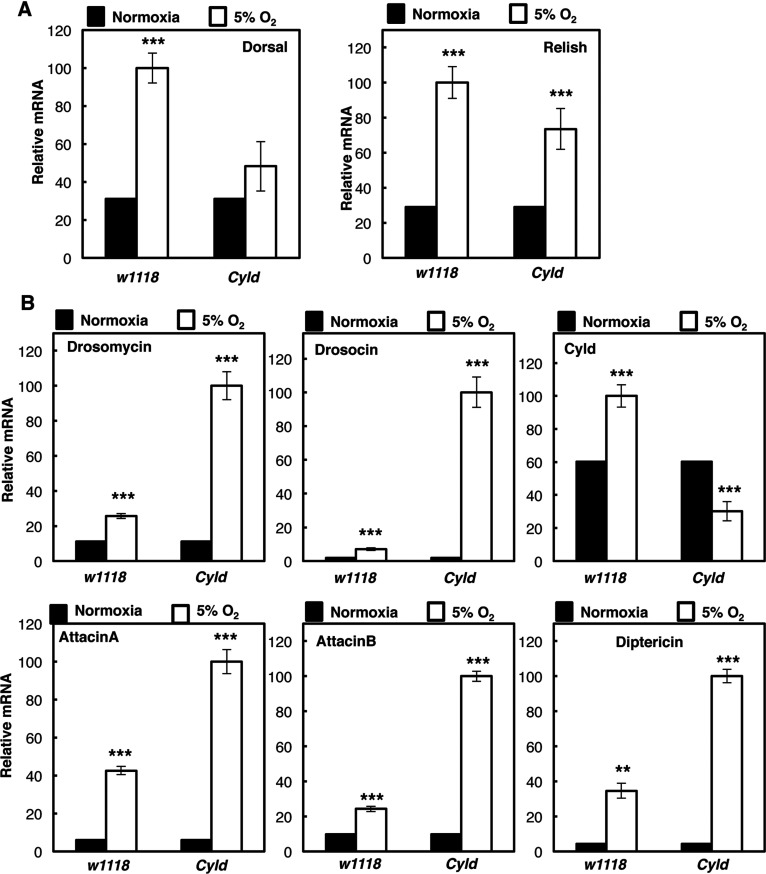
CYLD is a negative regulator of hypoxia induced NF-κB in *Drosophila* (**A**) Wild-type (*w1118*) and CYLD-deficient adult animals (*Cyld*) were exposed to 24 h 5% O_2_ prior to total RNA extraction. Levels of Dorsal and Relish mRNA were analysed by QPCR using actin as a normalizing gene. Graph depicts mean and S.E.M. of a minimum of three independent experiments. Student's *t* test was performed and significance determined as follows:*P≤ 0.05, **P≤0.01, ***P≤0.001. (**B**) Wild-type (*w1118*) and CYLD-deficient adult animals (*Cyld*) were exposed to 24 h 5% O_2_ prior to total RNA extraction. Levels of the indicated immune response genes were analysed as in (**A**). Graph depicts mean and S.E.M. of a minimum of three independent experiments. Student's *t* test was performed and significance determined as follows:*P≤0.05, **P≤0.01, ***P≤0.001.

An interesting observation from our analysis of hypoxia induced NF-κB in *Drosophila*, was the induction of Cyld. Cyld is a tumour suppressor gene and a known negative regulator of NF-κB [18]. We used *Cyld* loss-of-function *Drosophila* strains to investigate its role in the NF-κB response to hypoxia. Although the NF-κB subunit *relish* was unaffected by the presence or absence of *cyld*, *dorsal* induction following hypoxia exposure was reduced, indicating that Cyld is required for the up-regulation of this gene in hypoxia (Figure 5A). Interestingly, most of the NF-κB targets we have analysed, were all significantly up-regulated in hypoxia, when the Cyld function was impaired (Figure 5B). These results suggest, that Cyld controls NF-κB activation in hypoxia much like it does in response to infection [27] in this model organism.

### Controlled NF-κB activity is required for survival in response to hypoxia

**Figure 6:**
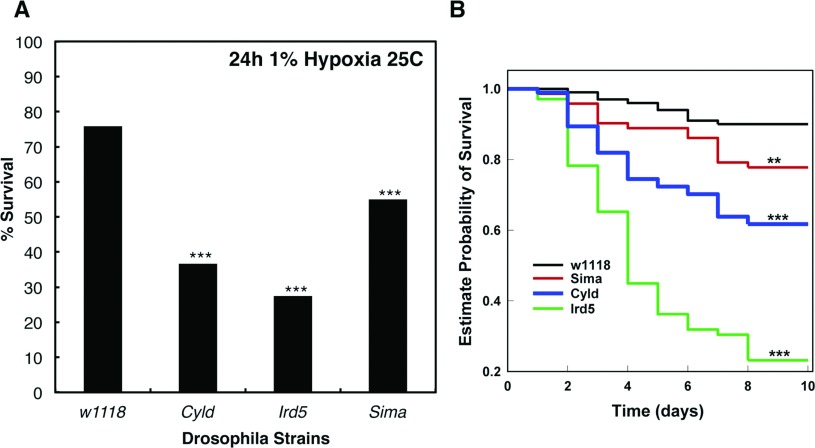
Precise control of NF-κB activity is required for *Drosophila* survival in hypoxia (**A**) Wild-type (*w1118*), IKK-deficient (*Idr5*), CYLD-deficient (*Cyld*) and HIF-deficient (*sima^07607^*) adult animals were exposed to 1% O_2_ for 24 h and survival quantified. Graph depicts percentage of survival of a total of 120 animals per strain. Student's *t* test was performed and significance determined as follows:*P≤0.05, **P≤0.01, ***P≤0.001. (**B**) Wild-type (*w1118*), IKK-deficient (*Idr5*), CYLD-deficient (*Cyld*) and HIF-deficient (*sima^07607^*) adult animals were exposed to 3% O_2_ and survival quantified. Groups of 60–80 flies were used. Survival was monitored and expressed as estimated probability of Survival. *P*-value was obtained from log-Rank statistical analysis.

All of our results so far, have implicated IKK and NF-κB in the response to hypoxia in *Drosophila*. To determine the biological consequences of this requirement, we performed viability measurements using the different *Drosophila* loss-of-function strains in response to hypoxia (Figure 6). We exposed adult animals to 1% O_2_ and measured survival in an acute response. As a control, we used a loss of function strain for the HIF-α homologue in *Drosophila*, Sima [22]. Following 24 h of exposure to 1% O_2_, *sima* deficient animals had a significant reduction in survival (Figure 6A). Interestingly, when NF-κB activity is either too low (*Ird5*) or too high (*Cyld*), survival is markedly reduced in hypoxia, suggesting that precise control of NF-κB is required for a proper response to hypoxia. We repeated this analysis under milder hypoxia conditions, 3% O_2_. Under these conditions, the survival is much more prolonged and wild-type animals survive relatively well for several days (Figure 6B). However, *Ird5* loss-of-function animals succumb quite rapidly to hypoxia exposure, even under these milder conditions, where *sima* mutant flies survive to about 75% following 10 days in hypoxia (Figure 6B). Interestingly, *Cyld* loss-of-function animals also survive less than both wild-type and *sima* loss-of-function strains indicating that precise control of NF-κB and the immune response in hypoxia, is required for survival of these organisms.

## DISCUSSION

In this report, we have investigated how hypoxia impacts on NF-κB in *D. melanogaster*. We have found that hypoxia activates NF-κB subunits, transcriptional activity and the immune response in the context of a larvae or adult fly. In addition, we demonstrate the requirement of the IKK homologue Ird5, for hypoxia-induced NF-κB, and the involvement of the tumour suppressor Cyld, in the negative regulation of this response.

*Drosophila* has been used extensively to delineate the innate immune response to bacteria, virus and fungal infection [19,28]. In the fly, innate immune responses can be activated via the Toll or IMD pathways [19,28]. These culminate in the release of an active NF-κB transcription factor, which in *Drosophila* comprises Dorsal, Dif and Relish [19,28]. Like in mammalian systems, the immune response also requires the activity of the TAK1 homologue [29]. In addition, the negative regulator, tumour suppressor and deubiquitinase Cyld, has also been associated with the control of the immune response in *Drosophila* [21].

In mammalian systems, hypoxia activates additional transcription factors [30,31], including NF-κB. Our own work, has investigated the mechanism behind NF-κB activation in mammalian cells [24,25]. We have identified the requirement of TAK1 and IKK for hypoxia induced NF-κB in mammalian cells [24,25]. The findings of the present study, demonstrate the crucial importance of Ird5 and NF-κB for the activation of the immune response and importantly survival in hypoxia. This also demonstrates that hypoxia-induced NF-κB is conserved from humans to fruit flies, as NF-κB has been previously shown to be required for survival of mammalian immune cells in hypoxia [32].

Transcriptional analysis of hypoxia-induced responses had previously identified immune genes as being overrepresented in the datasets analysed [33]. As such, our analysis is in agreement with these generic transcriptomic analyses. However, it is not clear why low oxygen would activate the immune response. It is likely that in conditions of infection, low oxygen regions are present and this would increase the immune response, as hypoxia and inflammation are often associated with each other [34]. This could represent an adaptation response of the immune system.

Our results also identify Cyld as a negative regulator of NF-κB activation in hypoxia. Cyld is a tumour suppressor, with roles controlling cell death, cell cycle and immune responses [18]. In mammalian systems, Cyld is a NF-κB target [35] and represses NF-κB in a negative feedback mechanism by its action on IKKγ and adaptor molecules such as TRAF2 and TRAF6 [18]. Thus far, Cyld role in the mammalian response to hypoxia has not been investigated in detail. One study demonstrated that Cyld is targeted for degradation by HPV (human papillomavirus) E6 virus, to prolong NF-κB activation following hypoxia [36]. In *Drosophila*, Cyld has been previously shown to interact with the IKKγ homologue (Kenny) and restrict NF-κB's activity both in basal and following bacterial infection, via the IMD pathway [27]. Our results are in agreement with this finding, as hypoxia activates mainly the IMD pathway in *Drosophila*. Our results thus identify Cyld as a component of the hypoxia response in *Drosophila*.

In mammalian systems, hypoxia has been shown to induce calcium release and subsequent activation of the TAK1 kinase complex [24]. Whether this also happens in *Drosophila* is not known, and requires further work. In addition, some studies have indicated that PHDs and FIH could control NF-κB activation [37–40]. However, the exact mechanism is still under debate, while some studies suggest a requirement for hydroxylase activity, others suggest that this effect is hydroxylase independent. In *Drosophila*, there is a single PHD gene, called Fatiga [22], while no FIH isoform has been identified. Unfortunately, the loss-of-function strain for *fatiga* is embryonic lethal [22] and hence the role of this enzyme in hypoxia induced NF-κB cannot be easily tested. In addition, the putative PHD-dependent hydroxylation motif is not present in Ird5/dIKK, suggesting at least that this is not through this motif that Fatiga could interfere with IKK function in the fly. However, we cannot rule out this possibility entirely thus far.

Unlike the majority of the mammalian cell system analysed, in *Drosophila* hypoxia activates the transcription of HIF isoforms Sima [23,41] and Tango [23]. Our previous work has indicated that in adult flies this requires the NF-κB subunit Dorsal [23]. Here we also demonstrate the requirement of the Ird5 kinase. As such, it is likely that Sima is regulated by a heterodimer Dorsal/Relish. In mammals, HIF-1α and HIF-1β can be directly regulated at their promoters by NF-κB heterodimers such as RelA/p50 [23,42–44], suggesting an extensive degree of conservation in this regulatory mechanism.

Overall, our study highlights how *Drosophila* would be a good genetic model to investigate hypoxia and inflammation. In the future, it would be interesting to determine tissue-specific responses and take advantage of the genetic tools available to further dissect the connection between these two conditions, so often present in human pathologies.
